# Impact of Storage Temperature on Prevalence of Adverse Events in Nasal Saline Sprays: Exploratory Analysis of Post-market Clinical Follow-Up Data

**DOI:** 10.7759/cureus.109873

**Published:** 2026-05-29

**Authors:** Mathieu M Albasser, Olga Reif, Edwin Sanchez, Abhay Aher, Abdul Haye, Petr Novak

**Affiliations:** 1 Clinical Research, Haleon Schweiz AG, Nyon, CHE; 2 Global OTC R and D Medical and Scientific Affairs, Haleon Germany GmbH, München, DEU; 3 Biostatistics and Data Management, Haleon US Inc., Warren, USA; 4 Therapeutics, Knowledge Centre, WNS Global Services, Gurugram, IND; 5 Global OTC Medical and Scientific Affairs, Haleon Czech Republic s.r.o., Prague, CZE

**Keywords:** adverse events (aes), nasal saline sprays, otrisal, otrivin sea water, post-market clinical follow-up (pmcf), prorhinel, storage temperature

## Abstract

Introduction

Nasal saline sprays are widely used for nasal hygiene and symptomatic relief in upper respiratory conditions. While nasal saline sprays are generally safe and well tolerated, the impact of storage conditions (particularly temperature) on their safety has not been evaluated. This post hoc exploratory analysis of data from three Post-Market Clinical Follow-Up (PMCF) studies investigated the association of storage temperature and prevalence of user-reported adverse events (AEs) across four nasal sprays: Otrivin Sea Water (OSW), Otrivin Sea Water with Aloe Vera (OSWAV), Otrisal 0.74% NaCl Metered-Dose Spray (MDS), and Prorhinel (Haleon plc, Weybridge, UK).

Methods

The analysis included all subjects from the Modified Intention to Treat (mITT) population across three pooled PMCF studies that evaluated the percentage of subjects who stored the sprays at room temperature (RT) (20-22 °C (68-72 °F)), at temperatures above RT (> 20-22 °C (68-72 °F)), and below RT (4-20 °C (40-68 °F)) and reported AEs. Odds ratios were calculated to assess the impact of storage temperature on the prevalence of AEs.

Results

Storage outside the RT was associated with increased risk of AEs across nasal saline spray products. The odds of experiencing an AE were approximately 8.94 times higher for OSW, 4.48 times higher for OSWAV, and 4.32 times higher for Prorhinel when stored outside RT, compared with storage within RT. When combined, the Bag of Value (BoV) products (OSW + OSWAV + Prorhinel) showed an overall odds ratio of 4.85 for the occurrence of AEs. Notably, storage above RT was linked to the highest AEs risk, with odds ratios of 15.32 and 14.87 for OSW and OSWAV, respectively. However, no AEs were reported in subjects when Otrisal MDS was stored outside RT.

Conclusion

This analysis demonstrated a significant correlation between storage temperatures outside RT and increased rates of AEs. These findings reinforce the current guidance provided in the instructions for use of nasal sprays, supporting RT as the optimal in-use temperature to ensure product safety.

## Introduction

Nasal saline sprays are widely used for maintaining nasal hygiene, symptomatic relief of rhinitis, and supporting overall upper respiratory health. These are non-pharmacological interventions, typically composed of isotonic or hypertonic saline, recommended for daily use to flush out allergens, pollutants, and pathogens from the nasal cavity, thereby reducing congestion, dryness, excess mucus, and irritation [1]. Their safety profile is well-documented, with minimal adverse events (AEs) [2,3]. Despite general safety, their performance can be influenced by environmental factors, particularly storage temperature. Temperature fluctuations, whether due to climate change or improper storage, can affect the physical and chemical characteristics of formulations, potentially leading to changes in viscosity, particle size distribution, and microbial stability [4], all of which can impact their safety and efficacy [5]. This is especially relevant for products using the Bag-On-Valve (BoV) technology, which, while offering advantages such as product purity and extended shelf life, may also exhibit sensitivity to temperature extremes due to the pressurized nature of the system [6].

Although AEs are routinely monitored through post-market surveillance, the specific impact of storage temperature on the prevalence of AEs in nasal saline sprays remains underexplored. Existing literature lacks comprehensive data linking temperature deviations to increased AE rates. This gap is significant given the widespread use of these products in varying climates and storage conditions. The label instructions of nasal saline sprays usually provide storage conditions (between 0°C-30°C), driven by the product stability requirements through its shelf life [7,8]. A possibility that consumers may understand the on-label storage temperature instruction as the product in-use temperature cannot be ruled out [9]. This may be of clinical relevance in cases when the nasal sprays are stored either below or above the room temperature (RT) and may have the potential to elicit strong pain signals when applied to the nasopharynx [10].

To address this, this research conducted a post hoc exploratory analysis of three pooled Post-Market Clinical Follow-up (PMCF) studies for four nasal sprays: Otrivin Sea Water (OSW), Otrivin Sea Water with Aloe Vera (OSWAV), Otrisal 0.74% NaCl Metered-Dose Spray (MDS), Prorhinel, and the combined BoV products (OSW + OSWAV + Prorhinel) (Haleon plc, Weybridge, UK). By synthesizing real-world data from multiple PMCF studies, this analysis aimed to provide evidence-based insights into how temperature-related storage conditions may influence the safety profile of nasal saline sprays, with implications for product labeling, storage, and patient education.

## Materials and methods

Study design

This post hoc exploratory analysis combined data from three completed PMCF studies (Study 1: 217004, Study 2: 218234, and Study 3: 218285) to assess the prevalence of AEs reported by subjects in relation to storage temperatures for nasal saline sprays. This post-hoc analysis is exploratory in nature, as it is based on pooled data from studies not originally designed to evaluate the impact of storage conditions, with inherent limitations in confounder adjustment and variable standardization. Subjects were categorized based on the type of nasal saline sprays and storage temperature conditions. Adverse events (AEs) were defined according to each study protocol and included any unfavorable medical occurrence reported during the study period, irrespective of causal relationship to device use; these were harmonized into a unified dataset using consistent coding definitions. The primary outcome was the occurrence of at least one AE per subject.

Inclusion and exclusion criteria

Inclusion criteria were consistent across studies and focused on subjects with complete AEs reporting, and storage condition data were included. Subjects with incomplete AEs reporting and storage condition data were excluded.

Interventions (saline nasal sprays)

The following nasal sprays were involved in this study: OSW Nasal Spray, OSWAV Nasal Spray, Otrisal MDS, and Prorhinel spray infants-young children/Prorhinel spray infants-children (Baby nozzle), Prorhinel spray children-adults/Prorhinel spray adults (Regular nozzle), and Prorhinel spray jet tonic adults (Jet nozzle).

Data collection

Data were collected from PMCF studies evaluating the proportion of subjects who stored the sprays at RT (20-22 °C (68-72 °F)), above RT (> 20-22 °C (68-72 °F)), and below RT (4-20 °C (40-68 °F)). Additional questions on storage of the sprays were also collected to understand the real-life patient behavior related to the spray’s storage temperature and usage habits. Storage temperature conditions were categorized based on recorded or reported storage practices within each study and aligned into common categories (e.g., recommended vs. elevated temperatures) to enable comparability.

Statistical analysis

The SAS 9.4 software (SAS Institute, Cary, USA) was used for statistical analysis. A Cochran-Mantel-Haenszel test was performed to determine if there was a relationship between the prevalence of AEs and storage conditions. A relationship was said to exist if the resulting chi-squared p-value was less than the significance threshold α = 0.05. If such a relationship exists, an odds ratio and 95% confidence interval (CI) were computed to show the relative rate of AEs to storage conditions. The exact one-sided upper 95% CI was computed based on the Clopper-Pearson method.

For the primary analysis, a 2x2 contingency table was created to compare RT storage with combined above and below RT conditions, and the specified statistical methods were applied accordingly. For the secondary analysis, a 2x2 contingency table comparing i) RT vs above RT, ii) RT vs below RT, and iii) above RT vs below RT was created to present subgroup comparisons, and the statistical methods described earlier, such as odds ratio calculation and confidence interval estimation, were applied accordingly.

## Results

PMCF study population and subgroups/stratifications

This study included data from 2,137 subjects across three PMCF studies. The sample comprised 1,027 subjects from the study on OSW and OSWAV (Study 1), 555 subjects from the Otrisal MDS study (Study 2), and 555 subjects from the Prorhinel study (Study 3). Subjects were categorized based on reported AEs and their corresponding product storage temperatures, viz., below RT, above RT, at RT, and a combined group of above and below RTs. These categories were further stratified by product type, including OSW, OSWAV, their combination (OSW + OSWAV), Otrisal MDS, Prorhinel, and the combined BoV products (OSW + OSWAV + Prorhinel).

**Figure 1:**
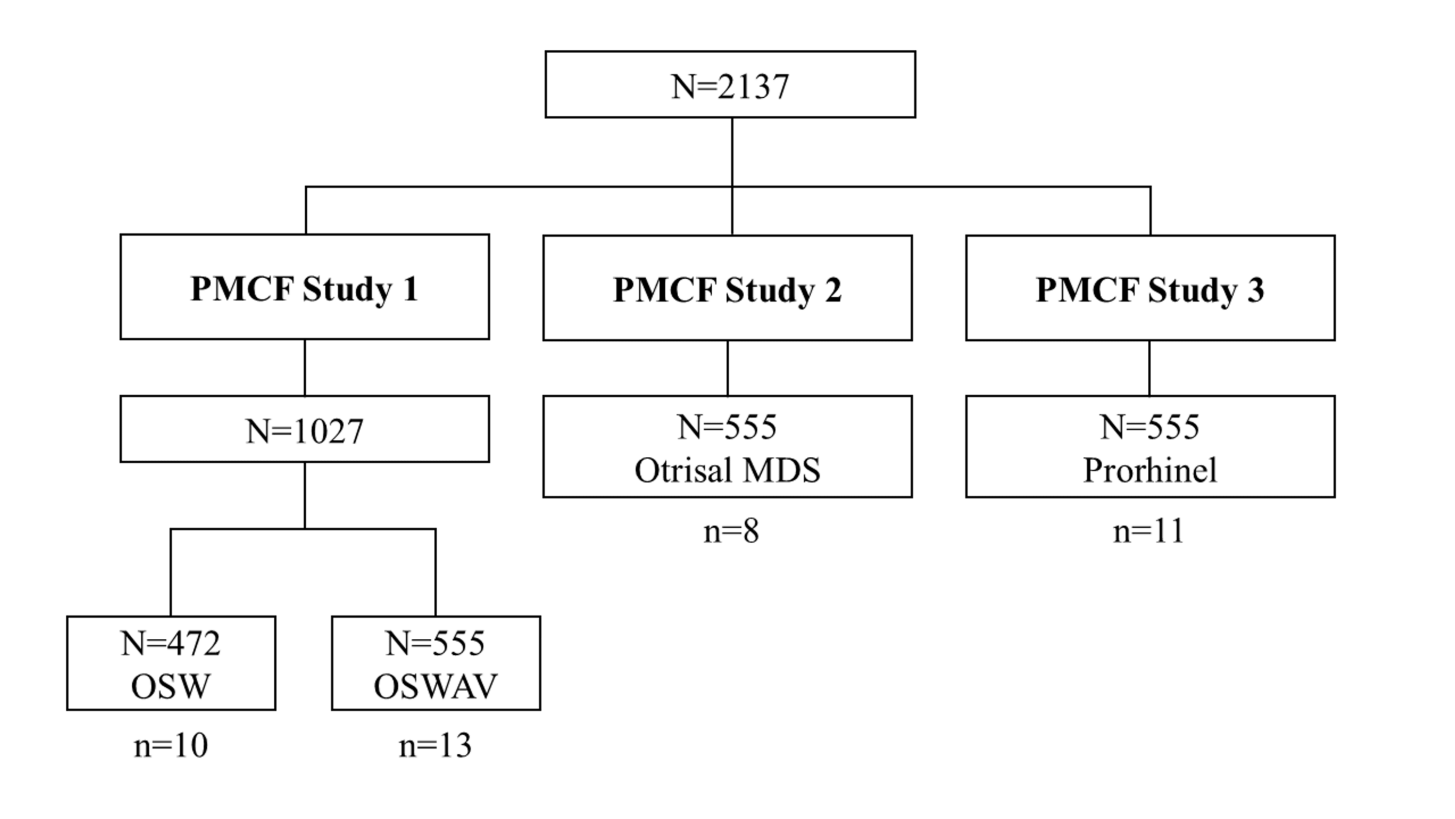
Participants disposition across PMCF studies. OSW, OSWAV, Otrisal MDS, and Prorhinel brands are owned by Haleon plc, Weybridge, UK. N: number of total participants in PMCF study, n: number of participants reported AEs. OSW: Otrivin Sea Water, OSWAV: Otrivin Sea Water with Aloe Vera, MDS: metered-dose spray, AE:m adverse event, PMCF: Post-Market Clinical Follow-Up

In total, 42 subjects across three PMCF studies have previously reported AEs, side effects (SEs), and/or device malfunctions (DMs) within the past six months. In the study involving OSW and OSW AV, 23 subjects (2.1%; 95% CI: 0.0%-2.9%) reported such events, with 10 subjects from the OSW arm (1.8%; 95 % CI: 0.0%-3.0%) and 13 subjects from the OSW AV arm (2.3%; 95% CI: 0.0%-3.7%). The Otrisal MDS PMCF recorded eight subjects (1.4%; 95% CI: 0.0%-2.6%), while the Prorhinel PMCF reported 11 subjects (2.0%; 95% CI: 0.0%-3.3%) (Figure 1).

Effect of storage temperature on adverse events (AEs) rates in nasal saline sprays

Room Temperature vs Above and Below Room Temperature

Storage of nasal saline sprays outside the recommended RT was associated with a higher likelihood of AEs. Increased odds of AEs occurrence were observed for OSW, OSWAV, their combined analysis, Prorhinel, and for BoV‑based formulation, whereas no AEs were reported for Otrisal MDS (Table 1).

**Table 1:** Adverse event prevalence at room temperature vs non-room temperature (above or below) for nasal spray products. "-" denotes that odds ratios were not computed when there were no AEs reported for a product/combination. OSW, OSWAV, Otrisal MDS, and Prorhinel brands are owned by Haleon plc, Weybridge, UK. N: number of subjects; n: number of subjects with AEs; Prevalence (%): n/N. OSW: Otrivin Sea Water, OSWAV: Otrivin Sea Water with Aloe Vera, MDS: metered-dose spray, RT: room temperature, non-RT: above and below RT, AE: adverse event

Product Type	Odds Ratio	95% CI	N (RT)	n (%) (RT)	N (non-RT)	n (%) (non-RT)
OSW	8.94	[1.93, 41.49]	433	4 (0.9)	39	3 (7.7)
OSWAV	4.48	[1.18, 17.05]	518	10 (1.9)	37	3 (8.1)
OSW + OSWAV	5.74	[2.14, 15.39]	951	14 (1.5)	76	6 (7.9)
Otrisal MDS	-	-	489	8 (1.6)	66	0
Prorhinel	4.32	[1.29, 14.46]	462	6 (1.3)	93	5 (5.4)
OSW + OSWAV + Prorhinel	4.85	[2.28, 10.31]	1413	20 (1.4)	169	11 (6.5)

Room Temperature vs Above Room Temperature

When analyses were restricted to storage above RT, a pronounced increase in AEs rates was observed for OSW and OSWAV, both individually and in combined analyses. In contrast, no AEs were reported for Otrisal MDS and Prorhinel. BoV‑based formulation demonstrated an increase in AEs occurrence when stored above RT. The comparative values are provided in Table 2.

**Table 2:** Adverse event prevalence at room temperature vs above room temperature for nasal spray products. "-" denotes that odds ratios were not computed when there were no AEs reported for a product/combination. OSW, OSWAV, Otrisal MDS, and Prorhinel brands are owned by Haleon plc, Weybridge, UK. N: number of subjects; n: number of subjects with AEs; Prevalence (%): n/N. OSW: Otrivin Sea Water, OSWAV: Otrivin Sea Water with Aloe Vera, MDS: metered-dose spray, RT: room temperature, AE: adverse event

Product Type	Odds Ratio	95% CI	N (RT)	n (%) (RT)	N (Above-RT)	n (%) (Above -RT)
OSW	15.32	[1.51, 155.14]	433	4 (0.9)	8	1 (12.5)
OSWAV	13.85	[3.34, 57.42]	518	10 (1.9)	14	3 (21.4)
OSW + OSWAV	14.87	[4.46, 49.63]	951	14 (1.5)	22	4 (18.2)
Otrisal MDS	-		489	8 (1.6)	0	0
Prorhinel	-		462	6 (1.3)	1	0
OSW + OSWAV + Prorhinel	14.66	[4.57, 47.01]	1413	20 (1.4)	23	4 (17.4)

Room Temperature vs Below Room Temperature

Storage below RT was also associated with elevated AEs rates for selected nasal saline sprays, most notably OSW and Prorhinel, resulting in increased odds relative to RT storage. No AEs were reported for OSWAV or Otrisal MDS at below‑RT. The pooled BoV product analysis similarly showed higher AEs occurrence below RT compared with RT, although the magnitude of effect was less pronounced than that observed for above‑RT (Table 3).

**Table 3:** Adverse event prevalence at room temperature vs below room temperature for nasal spray products. "-" denotes that odds ratios were not computed when there were no AEs reported for a product/combination. OSW, OSWAV, Otrisal MDS, and Prorhinel brands are owned by Haleon plc, Weybridge, UK. N = number of subjects; n = number of subjects with AEs; Prevalence (%) = n/N. OSW: Otrivin Sea Water, OSWAV: Otrivin Sea Water with Aloe Vera, MDS: metered-dose spray, RT: room temperature, AE: adverse event

Product Type	Odds Ratio	95% CI	N (RT)	n (%) (RT)	N (Below-RT)	n (%) (Below-RT)
OSW	7.4	[1.30, 42.08]	433	4 (0.9)	31	2 (6.5)
OSWAV	-		518	10 (1.9)	23	0
OSW + OSWAV	2.57	[0.57, 11.63]	951	14 (1.5)	54	2 (3.7)
Otrisal MDS	-		489	8 (1.6)	66	0
Prorhinel	4.37	[1.30, 14.63]	462	6 (1.3)	92	5 (5.4)
OSW + OSWAV + Prorhinel	3.51	[1.46, 8.44]	1413	20 (1.4)	146	7 (4.8)

Above Room Temperature vs Below Room Temperature

Direct comparison between above‑RT and below‑RT storage indicated a higher relative risk of AEs, particularly for combined OSW + OSWAV analyses and for BoV products, demonstrating higher odds of AEs occurrence with above RT storage compared with below RT storage (Table 4).

**Table 4:** Prevalence of adverse events across storage conditions (above vs below room temperature). "-" denotes that odds ratios were not computed when there were no AEs reported for a product/combination. OSW, OSWAV, Otrisal MDS, and Prorhinel brands are owned by Haleon plc, Weybridge, UK. OSW: Otrivin Sea Water, OSWAV: Otrivin Sea Water with Aloe Vera, MDS: metered-dose spray, AE: adverse event

Product Type	Odds Ratio	95% CI
OSW	2.07	[0.16, 26.22]
OSWAV	-	
OSW + OSWAV	5.78	[0.97, 34.26]
Otrisal MDS	-	
Prorhinel	-	
OSW + OSWAV + Prorhinel	4.18	[1.12, 15.63]

## Discussion

Maintaining nasal saline sprays at room temperature before use is recommended to support patient comfort and consistent product performance. Although the product remains stable across a wider storage temperature range, sprays used after being stored outside room temperature were observed to be associated with higher rates of reported AE rates compared to those used within the optimal range. This study indicated an association between patient-reported storage outside the room temperature range and increased adverse event (AE) reporting across various nasal saline spray products, including OSW, OSWAV, Otrisal MDS, Prorhinel, and their combined BoV formulations (OSW + OSWAV + Prorhinel). These findings align with post-marketing surveillance data, showing that deviations from room temperature (especially elevated temperatures) can compromise product performance and increase AEs reporting [3]. In contrast, Otrisal MDS did not show an apparent association between storage temperature and AEs. Although there were reports of both non-room temperature storage and AEs, no individual reported both simultaneously, suggesting a lack of direct association in this subgroup. This may be attributed to MDS technology, which deploys significantly lower product volume (µL) compared to bag-on-valve (mL) [11,12]. 

The statistical analysis of the relative rate of AEs to OSW/OSWAV nasal sprays storage conditions indicated an association between storage temperature and AEs (p<0.001). Products stored below room temperature were 2.8 times more likely to trigger an AEs, while those stored above room temperature were 14 times more likely. Overall, deviation from room temperature increased the AEs risk up to 15-fold compared to proper storage (p<0.001). This observed pattern may be explained by the physiological response of the nasal mucosa to thermal stimuli. When nasal sprays are used immediately after being stored outside the room temperature range (without allowing them to equilibrate), patients may experience thermal discomfort due to activation of nasal thermoreceptors, particularly those innervated by the anterior ethmoidal branches of the trigeminal nerve. These thermosensitive receptors are known to mediate sensations such as burning, stinging, or pain, which are frequently reported as AEs [13,14].

Recent studies have mapped intranasal thermal sensitivity, showing topographical differences in thermal thresholds across nasal regions, with the anterior septum being particularly sensitive to warming stimuli. This regional variation aligns with the dense sensory innervation of the nasal mucosa by branches of the trigeminal nerve (fifth cranial nerve), which mediates thermal and nociceptive signaling [14]. Studies demonstrated differential expressions of thermosensitive transient receptor potential (TRP) channels, notably transient receptor potential vanilloid 1 (TRPV1) and transient receptor potential vanilloid 3 (TRPV3), within these regions. TRPV1 activates at temperatures approximately >42 °C and is abundantly expressed in trigeminal ganglion neurons, contributing to heat-induced nociception (burning, stinging, and tickling sensations) [15]. TRPV3, in contrast, responds to innocuous warmth at around 33 °C and exhibits higher expression in the anterior septum, potentially explaining its enhanced thermal sensitivity [16].

Transient receptor potential melastatin-8 (TRPM8) is triggered by cool temperatures below 28 °C, eliciting a feeling of freshness and cooling, while transient receptor potential ankyrin 1 (TRPA1) becomes active at temperatures below 17 °C, often associated with stinging and burning sensations [13,14]. This sensitivity correlates with psychophysical measures of trigeminal and olfactory function, reinforcing the role of thermal stimulation in triggering nociceptive responses [13]. The primary mechanism underlying the sensation of ample nasal airflow is activation of trigeminal “cool” thermoreceptors by mucosal cooling, with cold receptors responding maximally in the 10-15 °C range. Furthermore, short- and long-lasting thermal stimuli can induce nociceptive responses in the nasal mucosa. These responses are modulated by analgesics and are indicative of Aδ- and C-fiber activation, respectively, supporting the hypothesis that thermal discomfort from improperly stored sprays may contribute to increased AEs reporting [17].

The observed differences in AEs under varying temperature conditions may also be attributed to the impact of temperature on nasal spray formulations and delivery systems. Additionally, BoV systems are susceptible to pressure changes under heat, which can alter spray force and droplet size, affecting delivery accuracy and patient comfort. These changes may lead to thermal discomfort, especially when sprays are used immediately after being stored in warm environments, triggering sensations such as burning or stinging due to activation of nasal thermoreceptors [18-20]. Conversely, lower temperatures (below room temperature) may induce physical changes in spray characteristics, such as increased viscosity, altered droplet size, and reduced spray force, which can affect deposition patterns and mucosal interaction. Cold sprays may also provoke cold-induced mucosal responses, including rhinorrhea, nasal congestion, and stinging, due to activation of TRP ion channels and mast cell-mediated pathways in the nasal mucosa. These responses are part of the body’s defense mechanism against cold air exposure and can mimic or exacerbate AEs symptoms [21,22]. Together, these findings highlight the importance of maintaining optimal storage conditions for nasal sprays to preserve product performance and minimize patient discomfort.

A key strength of this study lies in the use of pooled PMCF data from multiple nasal spray products, enabling robust cross-product comparisons and enhancing the generalizability of findings. The real-world nature of the data adds ecological validity, reflecting actual patient experiences. Furthermore, the use of the exact one-sided upper 95% confidence interval (CI), computed using the Clopper-Pearson method, strengthens the statistical rigor of the analysis by providing a conservative and reliable estimate of AEs rates. However, this study has several limitations. These include the lack of data on AEs severity, which restricts interpretation of clinical impact; reliance on self-reported storage conditions, which may introduce recall bias or misclassification; and limited sample sizes in certain temperature subgroups, potentially affecting statistical power. This study is a post hoc exploratory analysis of pooled data from studies not originally designed to assess storage condition effects, limiting adjustment for confounders due to inconsistent data availability. Variations in study design and data collection may introduce heterogeneity, and storage temperature reporting was not controlled. Additionally, the low incidence of AEs limits statistical power. Further studies are warranted to build upon these findings, incorporating objective temperature monitoring and standardized clinical grading of AEs to improve accuracy and interpretability.

Nonetheless, these results reinforce the importance of clear labeling and patient education regarding proper storage and application conditions for nasal sprays. Emphasizing room temperature storage in the instructions for use can help minimize the risk of AEs and improve overall patient experience. Additionally, healthcare providers play a crucial role in counseling patients on the significance of maintaining recommended storage temperatures. These measures can be integrated into routine practice, and manufacturers and clinicians can work together to enhance product safety, optimize therapeutic outcomes, and reduce preventable discomfort associated with improper storage. However, these observations remain speculative within the context of this analysis, as product performance parameters were not directly measured.

## Conclusions

This exploratory study underscores the critical importance of adhering to recommended storage conditions for nasal saline sprays. The observed increase in AEs reporting when products are stored outside room temperature highlights not only the potential compromise in product integrity but also the physiological sensitivity of the nasal mucosa to thermal stimuli. While the study findings do not provide a causal link between storage conditions and nasal spray in-use temperature, they underscore the importance of clear labeling and patient guidance on optimal storage practices. By integrating clinical data, post-marketing surveillance, and mechanistic insights from neurophysiology, this analysis provides a good understanding of how environmental factors (especially temperature) can influence patient experience and safety outcomes of nasal saline sprays. These insights may inform future product design, labeling, and patient education strategies to minimize risk and enhance therapeutic efficacy. Future research conducted by the broader scientific community could strengthen the evidence and further explore these associations across similar devices. Such studies may help elucidate the mechanisms underlying rare serious adverse events reported with nasal applicator use, including events associated with both non‑medicated and medicated formulations that otherwise demonstrate favorable benefit-risk profiles.
